# Twenty-Two-Year Outcome of Cartilage Repair Surgery by Perichondrium Transplantation

**DOI:** 10.1177/1947603520958146

**Published:** 2020-09-15

**Authors:** Maarten P. F. Janssen, Esther G. M. van der Linden, Tim A. E. J. Boymans, Tim J. M. Welting, Lodewijk W. van Rhijn, Sjoerd K. Bulstra, Peter J. Emans

**Affiliations:** 1 Department of Orthopaedic Surgery, CAPHRI School for Public Health and Primary Care, Maastricht University Medical Center, Maastricht, Netherlands; 2 Department of Orthopaedics, University of Groningen, University Medical Center Groningen, Groningen, Netherlands

**Keywords:** articular cartilage, cartilage repair, knee, cartilage transplantation

## Abstract

**Objective:**

The main purpose of the present study was to assess the risk for major revision surgery after perichondrium transplantation (PT) at a minimum of 22 years postoperatively and to evaluate the influence of patient characteristics.

**Design:**

Primary outcome was treatment success or failure. Failure of PT was defined as revision surgery in which the transplant was removed, such as (unicondylar) knee arthroplasty or patellectomy. The functioning of nonfailed patients was evaluated using the International Knee Documentation Committee (IKDC) score. In addition, the influence of patient characteristics was evaluated.

**Results:**

Ninety knees in 88 patients, aged 16 to 55 years with symptomatic cartilage defects, were treated by PT. Eighty knees in 78 patients were eligible for analysis and 10 patients were lost to follow-up. Twenty-eight knees in 26 patients had undergone major revision surgery. Previous surgery and a longer time of symptoms prior to PT were significantly associated with an increased risk for failure of cartilage repair. Functioning of the remaining 52 patients and influence of patient characteristics was analyzed using their IKDC score. Their median IKDC score was 39.08, but a relatively young age at transplantation was associated with a higher IKDC score.

**Conclusions:**

This 22-year follow-up study of PT, with objective outcome parameters next to patient-reported outcome measurements in a unique group of patients, shows that overall 66% was without major revision surgery and patient characteristics also influence long-term outcome of cartilage repair surgery.

## Introduction

Articular cartilage is a specialized connective tissue that provides a low-friction surface in joints, enabling human movement.^1^ However, when damaged, articular cartilage has poor regenerative capacity. When left untreated, cartilage defects eventually catabolically predispose the affected joint for the development of osteoarthritis (OA).^2,3^ To be able to treat such cartilage defects, several different articular cartilage repair strategies like microfracture, osteochondral allograft transplantation, mosaicplasty, perichondrium transplantation (PT), autologous chondrocyte implantation, and scaffolds have been developed over the past decades.^4,5^ The aim of these techniques is to form hyaline-like cartilage to create a pain-free functioning of the joint and prevent or postpone the development of OA and subsequent joint replacement.^6-9^

Various factors are correlated with a positive outcome of cartilage repair surgery. Examples are younger age, short duration of symptoms,^10,11^ and no history of previous surgery on the knee.^12,13^ No consensus can be found in the literature on whether the defect location influences outcome, but the occurrence of multiple lesions in one joint is described to impair outcome.^10,14^ There has been a gain of knowledge over the years on articular cartilage repair strategies and the importance of adequate patient selection to improve surgical outcome.^14^ Therefore, several treatment algorithms were developed to aid in patient selection for cartilage repair surgery.^15-19^ However, these algorithms are mostly based on short- and medium-term clinical outcome of cartilage repair surgeries. To our knowledge, there are no algorithms based on objective outcome parameters such as major revision surgery on the long term.

From 1986 till 1992, 88 patients with symptomatic cartilage defects in 90 knees were treated by PT.^20^ After 1-year follow-up Homminga *et al*. showed that 18 out of 25 patients treated with PT were symptom-free and had resumed their previous work and activities.^21^ In 1997, Bouwmeester *et al*. published the 5-year follow-up results of this study. They described 48 treatment failures, although it should be noted that they applied strict criteria to define a failure: being a reoperation, any change in arthroscopic graft appearance or an Hospital for Special Surgery (HSS) knee score of <75.^20^ In 40 out of 88 patients, there was a fair to good outcome of the procedure (HSS knee score above 75 and 85, respectively, combined with a good graft appearance on arthroscopy).^20^ Improved short-term results were described in patients with a single defect, without previous debridement operations, a long history of symptoms, age over 40 years, and a grade 2 or worse OA.^20^ A follow-up study was published in 1999, which presented the histological and biochemical results of these transplants.^22^ Because the overall results were found unsatisfactory, PT was only sporadically performed after its introduction. However, the PT-treated patient group is unique because of the 22-year follow-up period, enabling us to analyze the outcome based not only on patient reported outcome measurements but also on objective parameters, such as revision surgery, over time. The aim of this study was to chart the long-term clinical outcome after 22 years of follow-up after PT and to examine whether patient selection also influences objective outcome parameters such as major revision surgery next to patient-reported outcome measurements in this type of cartilage repair surgery.

## Methods

### Perichondrium Transplantation Operative Technique

Perichondrium transplantation is a single stage open procedure with 2 operation sites. Complete study details and early findings were described by Homminga *et al*. and Bouwmeester *et al*. in 1990, 1997, 1999, and 2001.^20-23^ In short, as described by Bouwmeester *et al*. in 1997,^20^ the procedure starts with debridement of the articular cartilage lesion up to the subchondral bone and a sharp vertical edge will be created on the surrounding cartilage. An oblique incision will be made over the lower part of the left side of the chest. The fascia of the rectus muscle is split transversely and the muscle is split in the line of its fibers. A piece of perichondrium will be dissected from the cartilaginous part of one of the lower ribs and removed together with its chondrogenic layer. The graft will be cut to match the size of the lesion. The perichondrial graft is then placed into the lesion with the chondral side facing up and will be attached with human fibrin glue.^21^

### Patients

From September 1986 until December 1992, 90 knees with articular cartilage defects in 88 patients were enrolled in the study. Eligible patients included men and women aged 16 to 55 years with symptomatic cartilage defects of the femoral condyles, patella, or trochlea, who were treated by PT. No exclusion criteria other than age >55 years were used for surgery.

Patient information on preoperative and short-term postoperative pain and function was retrieved from previous studies for 88 patients (90 knees). Based on these data, we were able to contact 78 patients (80 knees). Five patients were deceased and 5 patients were unable or unwilling to cooperate. Other than those lost to follow-up (*n* = 10), no patients were excluded in this long-term study.

### Outcome Assessment

Adequately defining the outcome of cartilage repair surgery is hard because no consensus exists on what is successful or nonsuccessful. In previous literature, failure of cartilage repair surgery has been described ranging from no improvement on functional outcome scores to re-intervention in which the graft is removed.^24-27^

For the present study, 2 different groups were specified. The first group contained the patients who underwent major revision surgery in which the graft was removed and/or arthroplasty was performed. This group that underwent major revision surgery was defined as treatment failure. Shaving of the transplant was not classified as major revision surgery. Patients who underwent major revision surgery were not asked to complete any questionnaires because their results would reflect the effect of the major revision surgery rather than the effect of the PT. The time of the PT and the time of major revision surgery was known and thus the time to failure of the treatment could be calculated. A survival analysis was performed on these data and the influence of patient characteristics on the time to failure was assessed.

Unfortunately, data on preoperative pain and function was incomplete and could not be used reliably for comparison with our long-term follow-up IKDC score. Patient characteristics at time of surgery we assessed that might be of influence were based on available literature and those found by Bouwmeester *et al*. at 52-month follow-up.^10-14,20,25,26^ Patient age, sex, number of lesions, lesion size, previous surgery, duration of symptoms, location in the knee, and grade of OA were described. Preoperative degree of OA, location in the knee, and type of previous surgery were not included in the cox and linear regression analyses. Only 6 people had an arthroscopically graded Outerbridge OA score higher than grade 2 in other parts of the knee in this cohort at the time of surgery. Also, group sizes of location in the knee and type of previous surgery were too small for statistical analysis. To identify predictors of outcome, univariate Cox regression was performed on possibly important preoperative factors with the outcome being treatment failure. Parameters with a *P* value <0.100 were subsequently analyzed in a multivariate Cox regression analysis. Because a maximum of 2.8 (*n* = 28/10) characteristics may simultaneously be analyzed, an explorative analysis was performed and by stepwise regression excluding the factor with the highest *P* value until only characteristics with *P* values <0.05 were present.

The second group contained the patients without revision surgery. They were asked to complete the International Knee Documentation Committee (IKDC) questionnaire. The IKDC questionnaire is best suitable to depict overall functioning for this ageing patient population with a long-term follow-up.^28^ These data were analyzed by linear regression in a similar way. Missing data, caused by patients that failed to complete the questionnaire, were calculated and completed by stochastic regression imputation.

### Statistical Analysis

Patient characteristics are presented as medians with corresponding interquartile range (IQR) for numerical variables and as number of patients (*n* and %) for categorical ones. A Kaplan-Meier survival analysis was performed to provide insight in the time to failure for these patients. Hazard ratios (HR) were subsequently calculated using univariate and multivariate Cox regression analysis. Patients who did not undergo major revision surgery were defined as nonfailures and their clinical functioning was evaluated using the IKDC questionnaire. A simple linear regression was calculated to investigate the association between IKDC score and different patient characteristics. All analyses were conducted using a significance level of 0.05. Statistical analysis was performed using IBM SPSS statistics for Mac, version 25.

## Results

### Patient Characteristics

Eighty knees in 78 patients were eligible for analysis. The median follow-up time of this included cohort was 25 years (IQR 25-26 years) with a minimum of 22 years of follow-up. The median age at time of surgery was 31.5 years (IQR 23-39 years). The median age at follow-up was 56.5 years (IQR 48-64 years). Knee cartilage lesions were located on the medial femoral condyle, lateral femoral condyle, patella, and trochlea. The median lesion size was 3.0 cm^2^ (IQR 2.0-4.0 cm^2^). The median time of symptoms before index surgery was 36 months (IQR 24-60 months). Forty-four right and 36 left knees were treated in 47 men and 33 women (**Table 1**).

**Table 1.** Patient Characteristics of the 80 Knees in 78 Patients Included in the Follow-up Cohort^a^.

Patient Characteristics	*n* (%)	Median (IQR)
Age at surgery (years)		31.5 (23-39)
Age at follow-up (years)		56.5 (48-64)
Follow-up time (years)		25 (25-26)
Age <40 years	61 (76%)	
Age ≥40 years	19 (24%)	
Male knees	47 (59%)	
Female knees	33 (41%)	
Defect size (cm^2^)		3.0 (2.0-4.0)
Defect location		
Medial femoral condyle	26 (32.5%)	
Lateral femoral condyle	2 (2.5%)	
Patella/trochlea	36 (45%)	
Multiple	16 (20%)	
Time since onset symptoms (months)		36 (24-60)
Knee with previous surgery	61 (76%)	
Knee without previous surgery	19 (24%)	
Arthroscopic degree of osteoarthritis at surgery (Outerbridge classification)		
None (grade 0)	58 (72.5%)	
Little (grades 1-2)	16 (20%)	
Definite (grades 3-4)	6 (7.5%)	

*n* = number of knees; IQR = interquartile range.

a Values are described as a count and percentage of the total 80 knees or as a median with subsequent interquartile range.

### Outcome at 22-Year Follow-up

**Figure 1.**
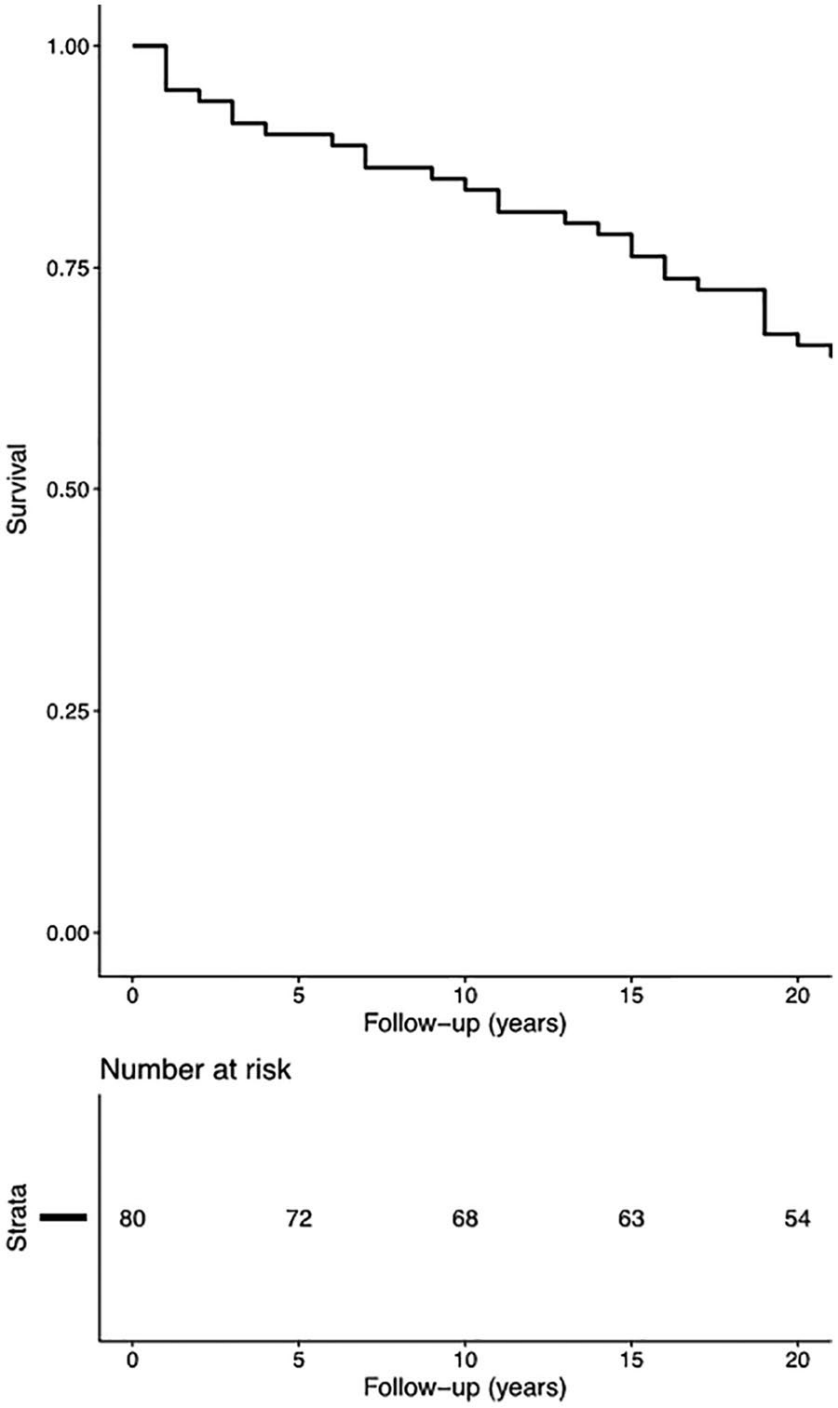
A Kaplan-Meier curve depicting graft survival (i.e., patients with no major revision surgery performed) up until the end of our current follow-up time of at least 22 years.

Twenty-six patients with 28 operated knees (35%) underwent surgery in which the transplant was removed. In 17 patients a total knee arthroplasty was performed, 2 patients underwent a patellofemoral arthroplasty, and 1 patient received a unicondylar arthroplasty. Also 6 patients underwent a patellectomy, which was used more frequently at that time as a salvage procedure. Finally, in 1 patient the transplant was removed. These surgeries were defined as major revision surgery and the treatment was classified as a failure. These failures occurred throughout the follow-up period of the study. A Kaplan-Meier survival analysis revealed that 95.0% was still without major revision surgery at 1 year (SE 2.4%), 83.8% at 10 years (SE 4.1%), and 66.3% at 20 years (SE 5.3%; **Fig. 1**).

### Influence of Patient Factors on Time-to-Failure of Treatment

A higher percentage (56%) of patients with multiple lesions treatment failed compared to patients with a single lesion (42%), HR 0.471 (0.213-1.043), *P* = 0.064. Treatment failed in 42% of the female patients and in 30% of the male patients, HR 1.602 (0.763-3.363), *P* = 0.213. In only 30% of patients younger than 40 years at the moment of primary surgery treatment failed versus 53% of patients older than 40 years at the moment of primary surgery, HR 0.487 (0.225-1.058), *P* = 0.069. In patients with a lesion size smaller than 3 cm^2^ 33% failed versus 38% in patients with a lesion size greater than 3 cm^2^, HR 1.166 (0.999-1.361), *P* = 0.051. In patients without previous surgery only 11% of treatments failed versus 43% in patients with previous surgery, HR 4.894 (1.161-20.642), *P* = 0.031, and in patients with symptoms shorter than 24 months there were less treatment failures compared to patients with symptoms longer than 24 months (15% vs. 45%, respectively), HR 1.011 (1.004-1.018), *P* = 0.001 (**Table 2**). These data was analyzed by univariate Cox regression analysis and subsequently explorative in a multivariate Cox regression analysis for characteristics with a *P* value <0.100 (duration of symptoms, previous surgery, size of the lesion, age at surgery, and surgery on multiple lesions) with the outcome being treatment failure and subsequent major revision surgery. Definite multivariate Cox regression was carried out with the characteristics “previous surgery” and “time of symptoms.” This definite multivariate Cox regression analysis showed that patients who were without previous knee surgery were significantly less at risk for treatment failure, HR 4.390 (95% confidence interval [CI] 1.036-18.598; *P* = 0.045). Subsequently, people with a shorter time from onset of symptoms until PT were significantly less at risk for major revision surgery, HR 1.010 (95% CI 1.003-1.017; *P* = 0.003). No significant differences were found for; size of the lesion, age at surgery, and number of lesions (**Table 2**).

**Table 2.** Overview of the Percentage of Failure of Perichondrium Transplantation in Different Patient Groups^a^.

	Number of Knees	Fail, *n* (%)	Univariate		Multivariate	
			Hazard Ratio (95% CI)	*P* Value	Hazard Ratio (95% CI)	*P* Value
Total	80	28 (35%)				
Number of lesions						
Single lesion	*64*	*19 (42%)*	0.471 (0.213-1.043)	0.064	*NA*	*NA*
Multiple lesions^b^	*16*	*9 (56%)*				
Patient age at time of surgery						
Age <40	*61*	*18 (30%)*	0.487 (0.225-1.058)	*0.069*	*NA*	*NA*
Age ≥40^b^	*19*	*10 (53%)*				
Lesion size						
Size of the lesion <3 cm^2b^	51	17 (33%)				
Size of the lesion ≥3 cm^2^	29	11 (38%)	1.166 (0.999-1.361)	*0.051*	*NA*	*NA*
Previous surgery						
Without previous surgery^b^	19	2 (11%)				
With previous surgery	61	26 (43%)	4.894 (1.161-20.642)	0.031*	4.390 (1.036-18.598)	0.045*
Duration of symptoms						
Duration of symptoms <24 months^b^	27	4 (15%)				
Duration of symptoms ≥24 months	53	24 (45%)	1.011 (1.004-1.018)	0.001*	1.010 (1.003-1.017)	0.003*

*n* = total number; % = percentage of the subgroup that failed; CI = confidence interval; NA = not applicable.

a Parameters with a *P* value <0.100 in univariate Cox regression analysis were subsequently analyzed in an explorative multivariate cox regression analysis (italic text) stepwise excluding characteristics with the highest *P* value and definite multivariate Cox regression analysis was performed on the characteristics, “previous surgery” and “time of symptoms” (plain text).

b Reference group.

* Significant influence.

### Influence of Patient Factors on Performance of Nonfailed Grafts at 22-Year Follow-up

Fifty-two PT patients (52 knees) were still without major revision surgery after a minimum follow-up of 22 years. To determine their functioning, these remaining patients were analyzed using the IKDC score. Their median IKDC score was 39.08 (IQR 25.57-53.74). Simple linear regression showed a significant relationship between IKDC and age at surgery (*P* = 0.012). No *P* values of <0.100 were found for other factors: number of lesions, previous surgery, time of symptoms, and size of the lesion. Therefore, no multivariate testing was performed on these data (**Table 3**).

**Table 3.** Univariate Linear Regression of Preoperative Factors That Possibly Correlate with the IKDC Score at 22 Years of Follow-up.

	B	95% CI	*P* Value
Number of lesions	8.163	−8.887 to 25.213	0.341
Age at surgery	−0.808	−1.428 to −0.187	0.012*
Size of the lesion	−2.496	−5.674 to 0.682	0.121
Previous surgery	−6.238	−18.632 to 6.156	0.317
Time of symptoms	−0.066	−0.257 to 0.126	0.494

IKDC = International Knee Documentation Committee; CI = confidence interval.

* Significant correlation.

## Discussion

The most important finding of this study was that after 22 years of follow-up of cartilage repair surgery in the knee by PT, 66% was still without major revision surgery. Duration of symptoms prior to surgery and previous surgery of the knee are predictors for undergoing major revision surgery and a younger age at primary cartilage repair surgery is associated with a better functioning as measured by IKDC. In the current literature, only limited studies are available with a long-term follow-up of cartilage repair surgery of the knee. Consequently, the outcome on the long term is mainly available by extrapolating short-term results,^24,29^ or in studies with relatively small group sizes.^30,31^

On a shorter follow-up term, Moradi *et al*., Krishnan *et al*., and de Windt *et al*. reported a higher patient age and a longer time of symptoms prior to cartilage repair surgery as a negative factor for successful outcome.^10,11,32^ Furthermore, Krishnan *et al*., Minas *et al*., and Pestka *et al*. found previous surgery of the knee as a negative factor for successful outcome.^10,12,13^ The follow-up time of many studies is too short for patients to reach an objective endpoint that defines treatment failure (i.e., OA, knee arthroplasty); therefore, published results are often based on patient-reported outcome measurements, and this can however lead to different forms of bias. Knee function deteriorates with increasing age and patient-reported outcome measurements, when not corrected for age, and can underestimate the outcome.^33,34^ Exceptions are the studies of Gobbi *et al*. who report increased osteoarthritic changes in older patients at 15 years of follow-up and the study of Minas *et al*., which did include knee arthroplasty, but with a 10-year follow-up period, *n* = 210, and 20 years, but with little patients left, *n* = 23.^13,30,35^ Our survival rate of 84% at 10-year follow-up is lower than the survival rate of 89% found by Gobbi *et al*. after microfracture.^35^ In contrast to this study we did not apply exclusion criteria (e.g., lesion size) other than age >55. The 79% survival rate reported of autologous chondrocyte implantation by Minas *et al*. is even lower, but this study treated patients with a larger average lesion size.^13^ The only comparison at 20-year follow-up can be made with the study of Ogura *et al*., who reported a survival rate of 63%, which is similar to our survival rate of 66%.^30^ Interestingly, this survival is already reported at their 10-year follow-up, but maintained in their 20-year follow-up. In general, our study has a comparable survival rate and confirms important patient characteristics, but after a longer-term follow-up, in a large patient group and with objective outcome measurements next to patient-reported outcome measurements.

A challenging aspect in cartilage surgery remains to define what treatment failure is. Definitions of failure in the current literature range from total knee arthroplasty or removal of the implant to a lack of improvement on questionnaires or Visual Analog Scales (VAS) for pain.^29^ This wide variety of definitions complicates an adequate comparison of different studies and can be a cause of the great differences in described predictors for success.^10-14,25-27,36,37^ Clinical functioning and quality of life is an important outcome factor, and therefore clinical questionnaires were included. However, with increasing age, knee function decreases. A deterioration of the IKDC score as described by Anderson *et al*.^38^ should therefore not be ignored. This is especially important in studies like this with a very long-term follow-up with an ageing population.^34^ Ideally a correction for age like the *z*-score would be calculated and used for a more valid comparison among individuals, but unfortunately the *z*-score can only be calculated up to the age of 65.^38^ When comparing the individuals younger than 65 in this study, the *z*-score did not differ between the different age groups 35 to 50 and 51 to 65 (*z* = −1.5 and −1.3, respectively, *P* value = 0.27). Thus, in this study, when corrected for age, the IKDC score is not worsened in the older patient age group (51-65) compared to the age group 35 to 50. Furthermore, age was also not found as a confounding factor in the multivariate regression analyses. Still, caution is advised in its interpretation.^34^

We conducted a longitudinal cohort study with 22 years of follow-up. However, a limitation of the present study was that some clinical data have been retrieved retrospectively, especially preoperative data and questionnaires were incomplete. Without complete preoperative scores, we considered a comparison with the VAS and HSS Knee Scores at a follow-up of 24 months not reliable and it was not the aim of this article.

## Conclusion

We present the long-term survival results of PT. In line with literature presenting mid-term follow-up, a smaller risk of total knee arthroplasty or other major revision surgeries was found in patients with a shorter time of symptoms prior to PT and without previous surgery of the knee. Subsequently a better functional outcome of the knee was found in patients operated at a relatively young age.
